# The negative association of the SARS-CoV-2 pandemic with the health of mother and child considering maternal childhood maltreatment

**DOI:** 10.1186/s40359-023-01327-8

**Published:** 2023-09-27

**Authors:** Franziska Köhler-Dauner, Manuela Dalhof (Gulde), Lara Hart, Ute Ziegenhain, Jörg M. Fegert

**Affiliations:** Department of Child and Adolescent Psychiatry/Psychotherapy, University Hospital of Ulm, Steinhövelstraße 5, 89075 Ulm, Germany

**Keywords:** SARS-CoV-2-pandemic, Childhood maltreatment (CM), Maternal psychosomatic health, Physical well-being, Maternal depression, Maternal somatic symptoms, Preventive isolation, School closures

## Abstract

**Background:**

Social distancing strategies during the SARS-CoV-2 pandemic have left families facing a variety of different constraints. Especially in this stressful time, children need a stable parental home to prevent developmental consequences. Additional risk factors such as maternal childhood maltreatment (CM) may affect mother’s psychosomatic health and children’s physical well-being in this period.

**Objective:**

It was aimed to analyze the associations between maternal CM, mother’s mental health, and children’s physical complaints during the SARS-CoV-2-pandemic.

**Method:**

Mothers of a well-documented birth cohort from a longitudinal study were included in this study. Psychosomatic health was assessed with the PHQ-D and children’s physical health with the GBB-KJ during the pandemic. *N* = 159 mothers completed the online survey. To describe the maternal CM, data from a longitudinal survey were used.

**Results:**

The calculation of three mediation analyses demonstrate that maternal depression symptoms (c-path: *β* = 0.10, *p* = .02; c’-path: *β* = 0.07, *p* = .13), somatic symptoms (c-path: *β* = 0.10, *p* = .02; c’-path: *β* = 0.07, *p* = .13) and psychosomatic symptoms (c-path: *β* = 0.10, *p* = .02; c’-path: *β* = 0.06, *p* = .19) fully mediate the relationship between CM and children’s physical health complaints.

**Conclusions:**

Maternal CM experiences seem to be one relevant risk factor during the pandemic and seem to influence the way in which parents deal with stressful situations and increase the risk for depressive symptoms. The present results highlight the importance to provide individually adjusted assistance to help the families to get through the pandemic.

## Introduction

For over a year, everyone in the world has been confronted with various challenges and restrictions due to the current pandemic. Especially, young families are faced with several challenges and measures. Young parents are being confronted with the difficulty of permanently finding new solutions to the problems that arise, from increasing pressure from recession or unemployment, school at home, the absence of social support, caring for grandparents to abrupt closures of schools, and childcare facilities as well as loss of community programs and advice of increasing physical distance [1–4]. Moreover, especially for the youngest society members, “social distancing” required by the pandemic raises significant challenges. In addition to the dangers of actual infection with the Covid-19-virus, the children were also under enormous psychological stress due to social isolation, lack of community activities, and learning delays caused by school closures [2]. Their lifestyle has totally changed because of the absence of face-to-face social interaction with friends, teachers, and other contact persons as well as home schooling instead of classroom teaching and the closure of leisure centers, churches, and other institutions. These changes leave many children in situations with limited emotional, social, and infrastructural support so that essential developmentally relevant parameters of everyday life break away.

Children that do not have a stable parental home may feel the cognitive, social, and emotional consequences that may be more profound and severe than currently anticipated. Studies showing that specifically young children depend on a stable and safe family environment during periods of stress and uncertainty [5]. The characteristics of periods of stress and uncertainty also define a crisis such as the current SARS-CoV-2 pandemic. Besides, previous economic recessions have shown that a family’s health is threatened by factors like a parental history of psychological stress, unemployment, decreasing income and excessive debt. This has been demonstrated in the context of increasing rates of various mental disorders, parental substance-related disorders, or suicidal behavior as well as a decrease in mental well-being behavior [2, 6–8].

In addition to that, a current study has shown that parental mental health and their perception of stress have been impacted by the health risks and fears associated with SARS-CoV-2 and that the children’s well-being is negatively affected by these changes as well [9]. Already under pre-pandemic normal conditions several studies have shown a strong association between parental mental health and children’s physical health. Maternal depression, for example, is a predictor for worse general physical health of their offspring during early childhood [10, 11], poorer growth and higher risk of diarrhea [12, 13]. It is also indicated that the brain development of children from depressive mothers may be seriously weakened, with implications for their own later physical health [14].

The importance of parental mental health is obvious. In truth, not all parents are able to be especially organized and stress-resistant in times of a pandemic in favor of providing a safe and protective environment for their children.

One risk factor that is further increased by the distress during the current pandemic is the previous experience of childhood maltreatment (CM). The experience of stressful early life experiences is indicated as a relevant predictor for increased parental mental health problems and the development of psychopathologies [15, 16] as well as somatic symptoms [17]. Concretely, experiences of CM are related to adverse mental health consequences, lower physical functioning, and poorer general health [18] as well as elevated prevalence rates of major depressive disorder (MDD) [19, 20]. Even in a meta-analysis it was shown that a history of CM led to an increased risk for the development of recurrent and persistent depressive episodes and to a generally reduced treatment outcome [21]. In addition, CM is associated with an array of somatic and physical problems like cardiovascular diseases, diabetes, as well as increased blood pressure [22]. Moreover, the experience of more than one CM subtype, multi-type maltreatment, led to an increased risk of mental health problems [23] and psychological symptoms [24]. Also, Steine et al. [25] reported that there is a dose–response relationship between cumulative CM scores and diverse self-reported symptoms, like depression and physical pain.

In addition, the effects of maternal CM may be passed on intergenerationally. It can affect for example the child brain development if the mother was exposed to CM [26]. Recent research demonstrates that individuals with their own experiences of CM seem particularly at risk to develop mental distress in less stressful times like a pandemic [27–31].

It may be concluded that the current pandemic constitutes a great challenge to coping with the pandemic-associated consequences, particularly for individuals that have experienced CM. In addition to that, it is supposed that the risk of developing depressive and somatic symptoms is increased compared to individuals with lower emotional burden due to the pandemic situation.

Bearing in mind that numerous findings confirm the link between the health of parents and their children [10–13], children of parents with CM seem particularly at risk of developing health problems themselves especially in times of a pandemic. Consequently, it is fundamental to understand the development pathway between the parental experiences of CM and their own as well as their children’s subsequent health and behavioral outcomes for the purpose of supporting positive child development, especially in the context of further stressors caused by the ongoing pandemic.

Therefore, the aim of this study was to analyze the interplay between maternal stressful early life experiences and the mothers’ and children’s health during the SARS-CoV-2-pandemic. It is hypothesized that mothers with own experiences of CM are more likely to report more severe depressive and somatic symptoms and that a lower quality of maternal mental health is related with increased physical complains of children during the SARS-CoV-2 pandemic. Furthermore, it is hypothesized that mothers’ mental health mediates the relationship between CM and children’s physical complaints.

The present results corroborate and expand on previous findings not only by examining the associations between these variables but by modelling their interplay in a mediation analysis. Understanding the mechanism behind the influence of maternal CM on their children’s health provides important information to derive further clinical and practical implications.

## Methods

### Study design

The data were collected in a prospective study design within the TransGen joint interdisciplinary project. In the joint project TransGen protective and risk factors were investigated regarding a transgenerational transmission of maternal maltreatment experiences by including psychological, biological, and social factors. It was performed in consonance with relevant guidelines and regulations and was approved by the Ethics Committee of Ulm University.

Throughout the child’s first years of life mother–child-dyads were accompanied and examined. All mothers and their children were recruited at the maternity unit of the Ulm University Hospital. To indicate maternal experiences of childhood maltreatment (CM) the investigation started with a screening one to three days postpartum (measurement time t0) using the German version of the Childhood Trauma Questionnaire (CTQ) [32, 33]. Afterwards, further surveys were conducted at additional measurement time points.

Moreover, all participating mothers at t0 were asked to take part in two similar online “SARS-CoV-2 pandemic surveys” in order to investigate the current stress load experienced by families over the time of the SARS-CoV-2-pandemic. The first survey was available from May 18th – July 31st, 2020, the second from March 1st – May 31st, 2021. The findings presented here relate only to the second survey, as a larger sample could be reached with this online survey.

### Participants

A total of 533 mother–child-dyads were recruited in the women’s hospital of the University Hospital of Ulm shortly after childbirth from October 2013 to December 2015. The following criterions were considered to decide if participants would be included in the study: sufficient knowledge of German, the mother’s age ≥ 18 years and a good state of health of child and mother. Criterions for exclusion were current or former drug or alcohol abuse by the mother, poor health of the mother (e.g., AIDS disease, hepatitis, etc.), severe mental illness of the mother as well as an extremely low birth weight of the child (less than 1500 g), serious birth complications or premature birth (less than 37 weeks of pregnancy). These factors may influence the mental and physical health of the mother–child-dyad from the beginning in such a way that it could not be justified to carry out this study under these conditions. Altogether 240 mothers provided written informed consent. For data assessment they were invited 3 months postpartum (t1: laboratory and home visit). In a further laboratory and home visit 158 mother–child-dyads participated when the child was around 12 months of age (t2) as well as around the child’s third birthday (t3). 159 participating mothers agreed to fill out the second online “SARS-CoV-2 pandemic survey” and completed the survey until the end of May 2021.

### Measures

#### Sociodemographic variables

The sociodemographic variables gender, age of the child, the age and level of education of the mother and changes in income were collected in the “SARS-CoV-2 pandemic survey”. Mothers’ level of education was investigated by the question “What is your highest level of education?”. To classify their level of education the mothers could choose from 5 different answer options. With the question “Has the income available in your household fallen by more than a quarter since the beginning of the crisis?” the changes in income were assessed. They were gathered by a binarily coded answer.

#### CTQ

The German short version of the Childhood Trauma Questionnaire [32, 33] is a standard tool in the form of a retrospective self-report questionnaire that was used to examine maternal CM. The tool records the experiences of physical and emotional neglect as well as physical, sexual, and emotional abuse before the age of 18 years [34]. Each subscale of the CTQ is assessed with five items on a 5-point Likert scale. Therefore, the range of the subscale scores is 5 to 25 and the sum scores range from “none” maltreatment experiences (25 points) over “minimal” to “extreme” maltreatment load (125 points). All CTQ subscales are summed up in one score which was developed as a measure for the maltreatment load [35].

#### PHQ-D

The Patient Health Questionnaire (PHQ-D) for patients is a valid and time-saving self-assessment tool that can be used for screening, assessing the severity and progress of psychological syndromes. The PHQ-D covers the following areas: modules on depression (PHQ-9), anxiety (GAD-7), somatic symptom severity (PHQ-15), panic disorder, binge eating disorder / bulimia nervosa, alcohol abuse and stress. The subscales of the PHQ-D reflect the current diagnostic criteria according to DSM-5 and ICD-10. The German version of the PHQ, the “Health Questionnaire for Patients (PHQ-D)”, has been extensively validated in various random samples and standard values are available [36]

The separate diagnostic modules of the PHQ can also be used individually [37]. For the verification of the hypotheses on which the manuscript is based, the modules depression (PHQ-9) and somatic symptoms (PHQ-15) were focused on. The depression module comprises 9 items. The sum of the scales “somatic symptoms” comprises the 15 most common physical complaints of outpatients. The somatoform module comprises 13 items from the somatoform module and two items from the depression module [38]. In this investigation the original answer options were modified so that all items are rated on a 3-point Likert scale: 1 (“not impaired”), 2 (“slightly impaired”), and 3 (“severely impaired”). The severity of the individual modules is calculated and used as a basis. Because it was expected that the two modules would highly correlate, the additional variable “psychosomatic” was created. For this the 9 items of the depression module and the 13 items from the somatoform module were summed up.

#### GBB-KJ

The Giessen Complaint Form for Children and Adolescents (GBB-KJ) [39] is a validated, multi-dimensional psychometric questionnaire for the comprehensive measurement of physical complaints in both self and external judgment in children and adolescents. The screening records the complaint scales (1) exhaustion, (2) stomach problems, (3) pain in the limbs, (4) circulatory problems and (5) cold symptoms. The 5 subscales are represented by 7 items, each of which is recorded on a 5-point Likert scale through the following answer options: 1 (“rarely”), 2 (“sometimes”), 3 (“often”), 4 (“continuously”), and 5 (“never”). The items of the five complaint scales are also aggregated to a total value of complaint pressure. The screening procedure exists in a long form (consisting of 59 items) and in a short form (consisting of 35 items). For the current evaluation, the version of the third-party judgment based on the mother of the child and the short form of the questionnaire were used.

### Statistical analyzes

Statistical analyzes were conducted in R, version 4.0.3 for Windows. Descriptive statistics were produced to examine the variables’ distributions. Data were assessed for normality graphically with normal probability plots and statistically with the Kolmogorov–Smirnov test. The normal probability plots and the results of the Kolmogorov–Smirnov-test revealed that none of the variables were normally distributed. However, according to Hayes [40], the procedure that is presently employed for the mediation analysis can be considered robust against violations of normal distribution as a statistical requirement.

Homoscedasticity of residuals and linearity were graphically assessed with residual scatterplots, which indicated some heteroscedasticity but no conspicuous problems for linearity.

Multicollinearity was tested by inspecting the variance inflation factor and tolerance, which were located within the limits acceptable for regression analysis.

Pearson correlations were computed between control and model variables in order to review their bivariate associations before conducting the mediation analyses. Three mediation analyses were calculated to test the hypotheses, with CM as the independent variable, children’s physical complaints as the dependent variable, and maternal mental health as the mediator variable. Since the mediational variables in the three models are latent variables, structural equation modeling (SEM) with the lavaan package [41] of the software R was used to test the three proposed single mediator mediation models. The Sobel-z-test was used to test the total, direct and indirect effects inferentially. Three mediation analyses were performed to test the total, direct and mediated effect of maternal CM on the children’s physical complaints during the pandemic via the maternal depressive symptomology during the pandemic (model 1), via maternal somatic symptomology during the pandemic (model 2) and via maternal psychosomatic symptomology during the pandemic (model 3).

## Results

### Descriptive analyzes

At the time of the second online “SARS-CoV-2-pandemic survey” the children’s age was between 5.48 and 7.64 years (*M* = 6.49 years, *SD* = 0.58 years) and the average age of the mothers was 40.13 years (*SD* = 3.93 years). Half of the children were boys (54.1%) and most mothers reported to be married or in a partnership (93.7%). The mothers had achieved a general high education exemption: university degree (67.3%); grammar school degree (10.7%); secondary school degree (18.2%); no secondary school degree (3.1%); other school qualification (0.6%). Only 5.0% of participants had indicated a decrease in income. All descriptive statistics are presented in Table 1.

**Table 1:** Descriptive statistics of control and model variables

Variable	M	SEM	SD	Median	Min	Max	Skew-ness	Kurto-sis	%
Age of children	6.49	0.05	0.58	6.46	5.48	7.64	0.08	-1.04	_
Age of mother	40.13	0.31	3.93	39.84	30.09	49.81	0.10	-0.15	_
Child is a boy	_	_	_	_	_	_	_	_	54.1
Mother in a partnership	_	_	_	_	_	_	_	_	93.7
University degree	_	_	_	_	_	_	_	_	67.3
Grammar school degree	_	_	_	_	_	_	_	_	10.7
Basic secondary school degree	_	_	_	_	_	_	_	_	18.2
No secondary school degree	_	_	_	_	_	_	_	_	3.1
Other school qualifications	_	_	_	_	_	_	_	_	0.6
Decrease in income	_	_	_	_	_	_	_	_	5.0
Maternal sum of CM	33.32	0.92	11.60	29.00	25.00	95.00	2.51	7.35	_
Maternal depression	13.49	0.29	3.71	13.00	9.00	25.00	0.70	-0.20	_
Maternal somatic symptoms	20.89	0.30	3.74	20.00	15.00	33.00	0.66	-0.05	_
Maternal psychosomatic	30.65	0.47	5.94	30.00	22.00	49.00	0.62	-0.36	_
Complaints of child	12.12	0.52	6.57	11.00	1.00	30.00	0.70	-0.10	_

Analysis of the histogram and descriptive data of the sum of CM showed a strong right skew. The median suggests that at least half of all mothers indicated a low degree of CM. The range between 25.00 and 95.00 and the average sum of CM (*M* = 33.32, *SD* = 11.60) supports this orientation of the distribution with only few extreme values. Eight outliers were detected inspecting the corresponding boxplot. As they were crucial for variability in the sample, they were not excluded.

The investigation of the histogram for the extent of maternal depression somatic symptoms and psychosomatic showed much variability in the data. The mean of maternal depression was 13.49 (*SD* = 3.71). Maternal somatic symptoms averaged 20.89 (*SD* = 3.74) and the mean of maternal psychosomatic was 30.65 (*SD* = 5.94). For maternal somatic symptoms and maternal psychosomatic an outlier above Q3 could be detected but did not substantially distort either distribution and was therefore not excluded. The graphical and descriptive analysis of all these three variables concerning maternal mental health revealed that a considerable number of values were located at the end of the lower range, but the distribution was not noteworthily right skewed.

The mean of complaints of child ranged between a minimum of 1.00 and a maximum of 30.00. Regarding this the mean of 12.12 (*SD* = 6.57) and the median of 11.00 illustrate that at least half of all participating children show a relatively low severity of physical complaints. For complaints of child an outlier above Q3 could be found but did not distort either distribution noteworthily. Hence, it was not excluded.

### Mediation analysis

Before conducting the mediation analysis, Pearson correlations between control and model variables were computed in order to review their bivariate associations. The results showed that none of the control variables were significantly related to the hypothesized predictor, mediator, and criterion of the mediation model. The zero-order correlations between the maternal sum of CM, maternal depression, maternal somatic symptoms, maternal psychosomatic and complaints of child were found to be significant (see Table 2).

**Table 2:** Pearson correlations/zero-order bivariate correlations between variables

Variable	1	2	3	4	5	6	7	8	9	10
1. Age of child	_									
2. Age of mother	.11	_								
3. Gender of child	-.06	-.09	_							
4. Education	-.09	.21^**^	-0.07	_						
5. Decrease in income	-.02	.07	0.04	.11	_					
6. Sum of CM	.11	.07	0.02	-.10	.08	_				
7. Depression	-.07	-.05	0.03	-.06	.00	.19^*^	_			
8. Somatic symptoms	-.13	-.10	0.04	-.04	.00	.18^*^	.76^***^	_		
9. Psychosomatic	-.11	-.09	0.05	-.05	.02	.21^**^	.92^***^	.94^***^	_	
10. Complaints of child	-.10	.12	0.01	.01	.03	.18^*^	.37^***^	.35^***^	.39^***^	_

*N* = 159

^*^p < .05

^**^p < .01

^***^p < .001

Depressive symptoms showed a high correlation with somatic symptoms of the mother, *r*(157) = 0.76, *p* < 0.001. Regarding this, it must be considered that the somatic somatoform module comprises two items of the depression module. Psychosomatic was found to highly correlate with depression, *r*(157) = 0.92, *p* < 0.001, and somatic symptoms, *r*(157) = 0.94, *p* < 0.001. For this it must be taken into account that the variable psychosomatic includes items that were also used as the base of the two variables depression and somatic symptoms.

Complaints of child showed a small correlation with maternal sum of CM, r(157) = 0.18, *p* < 0.05 and a moderate correlation with maternal depression, *r*(157) = 0.37, *p* < 0.001, maternal somatic symptoms, *r*(157) = 0.35, *p* < 0.001 and maternal psychosomatic, *r*(157) = 0.39, *p* < 0.001. A small correlation was found between the maternal sum of CM with maternal depression, *r*(157) = 0.19, *p* < 0.05, maternal somatic symptoms, *r*(157) = 0.18, *p* < 0.05, and maternal psychosomatic, *r*(157) = 0.21, *p* < 0.01. Therefore, a significant influence between the model variables could be derived and the mediation analysis could proceed.

### Maternal depression mediates the interplay between maternal CM and complaints of child

**Fig. 1:**
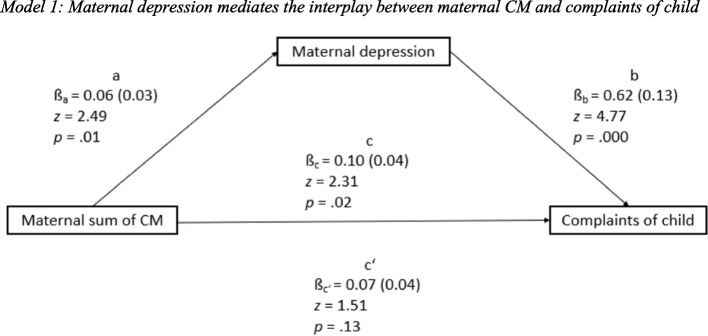
Model 1: Maternal depression mediates the interplay between maternal CM and complaints of child

The total (*p* = 0.02) and indirect (*p* = 0.03) effects were significant, the direct effect was not significant (*p* = 0.13). Unstandardized coefficients with corresponding standard errors, *z* values and *p* values as results of the mediation analysis are displayed in Fig. 1.

As indicated by the bivariate correlations, the maternal sum of CM was shown to significantly affect maternal depression (ß_a_ = 0.06, *SE* 0.03, *p* = 0.01). Hence, mothers with higher sums of CM were more likely to have depressive symptoms. Maternal depression further significantly predicted complaints of child, when controlling for the maternal sum of CM (ß_b_ = 0.62, *SE* 0.13, *p* = 0.000).

Maternal depression was observed to mediate the relationship between the maternal sum of CM and complaints of child significantly (indirect effect: ß_a*b_ = 0.04, *SE* 0.02, *z* = 2.21, *p* = 0.03). Though, under control of the mediator’s influence on the criterion, the direct influence of the maternal sum of CM on complaints of child did not remain significant with an effect of ß_c’_ = 0.07 (*SE* 0.04, *p* = 0.13). The total effect of the maternal sum of CM on complaints of child was ß_c_ = 0.10 (*SE* 0.04, *p* = 0.02). The direct effect no longer reached significance under statistical control of the mediator and the association was considerably reduced in size compared to the total effect. It seems that maternal depression is completely mediating the association between the sum of CM and complaints of child as the direct effect did not remain significant when including maternal depression as a mediator. Nevertheless, the fact that the total effect is significant, and the direct effect is not significant is insufficient to prove complete mediation.

All effects were controlled for gender and age of child, age and education of mother and decrease in income. The total and indirect effects remained significant and no significant influence of these could be identified (see Fig. 1).

### Maternal somatic symptoms mediate the interplay between maternal CM and complaints of child

**Fig. 2:**
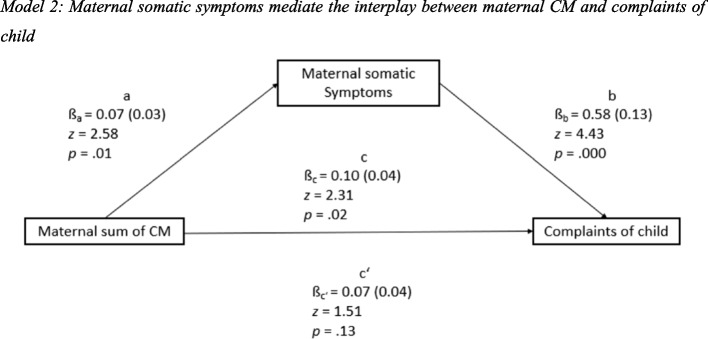
Model 2: Maternal somatic symptoms mediate the interplay between maternal CM and complaints of child

The total (*p* = 0.02) and indirect (*p* = 0.03) effects were significant, the direct effect was not significant (*p* = 0.13). The results of the mediation analysis, unstandardized coefficients with corresponding standard errors, *z* values and *p* values, are presented in Fig. 2.

As indicated by the bivariate correlations, the maternal sum of CM significantly predicted maternal somatic symptoms (ß_a_ = 0.07, *SE* 0.03, *p* = 0.01). Therefore, mothers with higher sums of CM tended to have more somatic symptoms. Additionally, maternal somatic symptoms significantly affected the frequency of complaints of child, when controlling for the maternal sum of CM (ß_b_ = 0.58, *SE* 0.13, *p* = 0.000).

Maternal somatic symptoms are identified as a significant mediator in the interplay between the maternal sum of CM and complaints of child (indirect effect: ß_a*b_ = 0.04, *SE* 0.02, *z* = 2.23, *p* = 0.03). However, when controlling for the mediator’s influence on the criterion, the direct influence of the maternal sum of CM on complaints of child did not remain significant with an effect of ß_c’_ = 0.07 (*SE* 0.04, *p* = 0.13). The total effect of the maternal sum of CM on complaints of child was ß_c_ = 0.10 (*SE* 0.04, *p* = 0.02). The results propose that the direct effect is decreased by the significant role of the mediator compared to the total effect. It appears that maternal somatic symptoms are completely mediating the relationship between the sum of CM and complaints of child as the direct effect did not remain significant when including the mediator. Nonetheless, a significance test is insufficient to prove complete mediation,

All effects were controlled for gender and age of child, age and education of mother and decrease in income. The total and indirect effects remained significant (see Fig. 2). The age of mother has been shown to influence complaints of child under control of the maternal sum of CM, the maternal somatic symptoms, and the other control variables (ß_c6_ = 0.26, *SE* 0.13, *z* = 2.07, *p* = 0.04). For the other control variables, no significant influence could be found.

### Maternal psychosomatic mediates the interplay between maternal CM and complaints of child

**Fig. 3:**
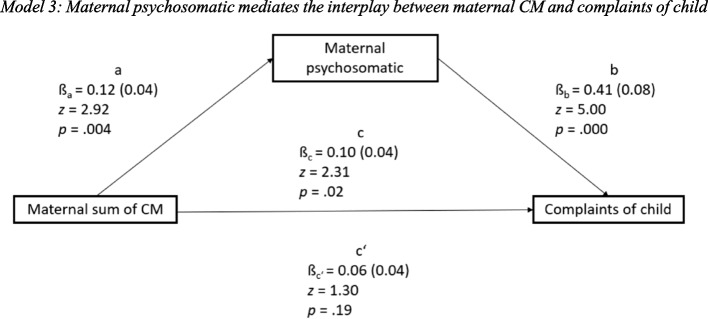
Model 3: Maternal psychosomatic mediates the interplay between maternal CM and complaints of child

The total (*p* = 0.02) and indirect (*p* = 0.01) effects were significant, the direct effect was not significant (*p* = 0.19). Unstandardized coefficients with corresponding standard errors, *z* values and *p* values as results of the mediation analysis can be found in Fig. 3.

As indicated by the bivariate correlations, the maternal sum of CM was shown to have a significant effect on maternal psychosomatic symptoms (ß_a_ = 0.12, SE 0.04, *p* = 0.004). Therefore, mothers with higher sums of CM tend to have more psychosomatic symptoms. Furthermore, maternal psychosomatic symptoms significantly affected complaints of child, when controlling for the maternal sum of CM (ß_b_ = 0.41, SE 0.08, *p* = 0.000).

The relation between the maternal sum of CM and complaints of child was significantly mediated by the maternal psychosomatic (indirect effect: ß_a*b_ = 0.05, SE 0.02, *z* = 2.52, *p* = 0.01). However, the direct influence of the maternal sum of CM on complaints of child did not remain significant under control of the mediator’s effect on complaints of child (ß_c’_ = 0.06, SE 0.04, *p* = 0.19). The total effect of the maternal sum of CM on complaints of child was ß_c_ = 0.10 (SE 0.04, *p* = 0.02). Under statistical control of the mediator the direct effect no longer reached significance. Additionally, the association was reduced in size compared to the total effect so that it appears that maternal psychosomatic is completely mediating the association between the sum of CM and complaints of child. However, as with model 1 and 2 the fact that the total effect is significant, and the direct effect is not significant is insufficient to prove complete mediation.

When controlling for gender and age of child, age and education of mother and decrease in income the total and indirect effects remained significant (see Fig. 2). The age of mother was shown to affect complaints of child under control of the maternal sum of CM, the maternal psychosomatic, and the other control variables (ß_c6_ = 0.25, SE 0.13, *z* = 2.04, *p* = 0.04). For the other control variables, no significant influence could be found.

## Discussion

From an attachment perspective, the current pandemic turned out to have far-reaching and exceptional implications for children, adolescents, and their families [42]. A lot of social-distancing restrictions were implemented into daily life to contain the spread of the SARS-CoV-2 virus, such as school closures and home schooling [43], quarantine, limited private shielding as well as the cancellation of out-of-home leisure time activities [44–46]. These constraints have added to the already existing burden on families, possibly to the point where their ability to cope with the associated challenges has been overburdened [2]. Moreover, consideration must be given to the fact that many institutional social systems and other family members failed to give the families external support anymore [2].

Current research is investigating the effects of the ongoing pandemic on children. Compared to older children, especially children between 3 and 6 years of age are considerably more likely to be affected by stress symptoms in their emotional and social development [47]. This emphasizes the significance of a stable and secure family environment. One essential component for this are mentally healthy parents, who serve for children as a strong protective factor in their daily life [5].

Therefore, the present study aimed to investigate for the first time maternal mental health under consideration of mothers’ history of CM by contrasting the effect of these factors on the children’s physical well-being during the SARS-CoV-2-pandemic. The results corroborate and expand on previous findings not only by examining the associations between these variables but by modelling their interplay in a mediation analysis. Understanding the mechanism behind the influence of maternal experiences of CM on their children’s physical health provides important information to derive further clinical and practical implications. Moreover, this study intends to provide insight into how a maternal history of CM significantly affects the mothers’ mental health and their children’s physical health. To put into context the appropriate variables during the pandemic, three models with three different maternal mental health variables were created, such as maternal depression, maternal somatic and psychosomatic health. In view of the findings of these preliminary analyses, a total effect of maternal CM on children’s physical health during the pandemic was expected. In addition to that the relation of maternal history of CM, the mothers’ mental health and their children’s physical complaints were examined. The relevance of this study considers the investigation of how the effect of mothers’ CM on their children’s physical health may work for the sake of understanding the underlying mechanism and to set up a framework for future research approaches. Before examining the results of the mediation analyses in more detail, first the present findings on the relationship between each of the model variables (mother’s CM experiences, mother’s depressive/somatic/psychosomatic symptoms and child’s physical symptoms during the pandemic) will be highlighted and considered in relation to previous research.

The results show that the extent of the mothers’ experienced CM was significantly related to the severity of depressive symptoms, of somatic symptoms and of psychosomatic symptoms experienced by mothers during the pandemic. These results are in line with several previous studies which indicate that adults with experiences of CM are more likely to develop mental health problems [27–31] as well as somatic and respectively psychosomatic symptoms [48–50]. For example, Newman and colleagues [48] found significantly higher numbers of somatic symptoms as well as physician visits among adults with childhood sexual abuse. Moreover, those adults who reported depressive symptoms in addition to their CM experiences were more likely to visit the emergency room than those without additional depressive symptomatology. Furthermore, existing findings clearly show that depressive symptoms are associated with stressful early life experiences [19, 49]. Also, childhood sexual abuse along with lack of adequate coping strategies have been found to be predictors of anxiety, trauma symptoms, somatic symptoms, and sexual dysfunction in young adults [49]. Thus, this result of the study can support the current state of research that CM experiences can be a risk factor for mental as well as health problems in later life. Moreover, because the symptom questionnaire explicitly refers to the current state of a pandemic, mothers with CM experiences seem to be a particularly vulnerable group for the development of such problems in times of crisis.

Further, the current findings complement existing findings in that it was shown that maternal mental as well as (psycho-)somatic health significantly predicted the physical complaints of their children. In this study it was found that the children’s physical health complaints during the pandemic significantly correlated with the maternal depression level, maternal somatic symptoms, and maternal psychosomatic symptoms. As mentioned in the theoretical derivation of the hypotheses, previous studies have already found an association between maternal mental health, especially depression, and child physical health [10–14]. In addition, an increased likelihood of a child having a chronic illness has been found when the mother has more psychiatric symptoms [51]. Similarly, in a cohort study of nearly 15,000 participants, maternal mental health, particularly anxiety and somatization, was found to be a determinant of the physical health status of their children [52]. In conclusion, the present finding supports the previous research on the relationship between maternal and child health accordingly.

Another finding of this study is the relationship between the mother’s CM experience and the child’s physical symptoms during the pandemic: A significant relationship between the maternal CM load and their children’s physical health during the pandemic was found. Since maternal CM experiences are associated with poorer mental and psychosomatic health, and this poorer maternal health has implications for the child’s physical health, this association seems plausible. Previous studies mostly focused on the transgenerational impact of maternal CM on children’s mental health [53, 54]. For example, in one study, approximately 22% of the variance in child Strengths and Difficulties Questionnaire scores could be explained by the presence of multistressors in the family and role reversal between parent and child [53]. Also, as early as 2005, Daud and colleagues [54] found an association between parents’ traumatic history and their children’s mental health: children of traumatized parents exhibited significantly more symptoms of anxiety, depression, posttraumatic stress, attention deficits, as well as conduct disorders [54]. However, the relationship between maternal CM and physical health of the child has not been studied so far, so this finding may add a new aspect to the existing knowledge.

Based on these associations, as well as existing literature, three mediation models were also tested to find out a possible explanatory factor for the higher physical complaints in children with increasing CM stress of their mothers. In this study, depressive, somatic and psychosomatic symptoms of the mother were investigated as possible mediators. Dixon and colleagues (2005) [55] point out that maternal psychopathology in particular is expected to be an important candidate for possible transmission of one’s own adverse childhood experiences to the next generation, especially during sensitive developmental windows such as may be presented by additional exposure to a pandemic [55]. This relationship was particularly well illuminated in the postpartum period. Since childhood abuse and neglect experiences were shown to be a particular risk factor for psychopathology later in life [20], it became particularly clear that this history has a great influence on the psychosomatic health of mothers, especially in the postpartum period, and that this health has a mediating effect on the mental health of the child [56, 57]. Furthermore, these findings have been confirmed in the later developmental age of the child [58, 59]. Also, Lamela and Figueiredo [50] previously demonstrated a mediation relationship between parents’ physical victimization and the risk of CM in their own children. This was mediated by the psychosomatic symptoms of the parents. Therefore, and due to the described associations above, the consideration of mental, somatic as well as psychosomatic symptoms as mediators appeared to be meaningful.

The results of the present mediation analyses of the first model propose that the mothers’ depressive symptomology significantly mediated the relationship between CM and children’s physical health during the pandemic. The total effect reached significance as expected based on the preliminary analyses, the direct effect did not. Therefore, during the pandemic, mothers that experienced more CM were at a significantly higher risk to have more severe depressive symptoms, which significantly contributed to their children’s physical complaints. Additionally, the mothers’ severity of depression was found to operate as a significant mediator between the mothers’ experience of CM and children’s physical health problems. During the pandemic, the maternal CM experience significantly predicted a higher level of depression-related symptoms, which was found to significantly raise the likelihood of children’s physical complaints, respectively.

In the second model the role of maternal somatic symptoms in the association between CM and children’s physical complaints was investigated. The outcomes of the mediation analyses of model 2 suggest that the mothers’ somatic symptomology significantly mediated the association between CM and children’s physical health during the pandemic. As expected, based on the initial analyses, the total effect was significant, the direct effect was not. Hence, during the pandemic, mothers that experienced more CM were at a significantly higher risk to have more severe somatic symptoms, which made a significant contribution to their children’s physical complaints. In addition to that, the mothers’ severity of somatic symptoms operates as a significant mediator between the mothers’ experience of CM and children’s physical health problems. During the pandemic, the maternal CM experience significantly predicted a higher level of somatic-related symptoms, which was found to significantly raise the likelihood of children’s physical complaints, respectively. In addition, when controlling for model variables, maternal age continued to be significantly related to child physical symptoms. This implies that as the mother’s age increases, children also experience more physical discomfort during the pandemic, which cannot be explained by CM experiences or increased somatic symptoms in the mother. It is possible that this can be explained by the fact that the mothers were already older at pregnancy and birth, and thus more likely to have complications and high-risk pregnancies.

In a final third model maternal depressive and somatic symptoms to psychosomatic symptoms were summarized and the role of this in the hypothesized associations was examined. It can be inferred from the results of the mediation analyses of model 3 that the mothers’ psychosomatic symptomology significantly mediated the association between CM and children’s physical health during the pandemic. This result is not surprising with regard to the significant models 1 and 2, since the recording of psychosomatic symptoms was done by summarizing the previously considered symptoms. However, since it was not clear when planning the analyses whether the separate consideration of psychological and somatic symptoms as explanatory factors would be sufficient, this value was also considered. As anticipated, based on the initial analyses, the total effect is significant, the direct effect is not. Consequently, during the pandemic, mothers that experienced more CM were at a significantly higher risk to have more severe psychosomatic symptoms, which contribute significantly to the physical complaints of their children. In addition to that, the mothers’ severity of psychosomatic symptoms operates as a significant mediator between the mothers’ experience of CM and children’s physical health problems. During the pandemic, the maternal CM experience significantly predicted an increased level of psychosomatic symptoms, which was found to significantly increase the likelihood of children’s physical complaints, respectively. Again, the mother’s age was positively significantly related to the child’s physical complaints beyond the model.

The outcomes of the three models corroborate the hypothesis that a maternal history of CM influences children’s subsequent physical health through the joint relation with the mothers’ depressive and somatic symptomology. Hence, they should be considered as further risk factors, not exclusive but especially in times of extensive stress.

The present study revealed a markedly differentiated reaction of families to the pandemic situation depending on the maternal CM history as well as partly the age of the mother. The results underscore the importance to meticulously register all risk factors possibly affecting the physical health of children and to analyze the interwoven systemic effects as this is the only way to offer an effective, tailored support in a pandemic crisis like the current one to the families with their unique needs and backgrounds. An especially well investigated example for such a latent background factor is the vicious circle of transgenerational transmission of maternal childhood maltreatment, maternal health problems and resulting physical health impairments of the children. It is essential to break such automatic mechanisms. The current results indicate that supporting mothers with a history of CM and concurrent depressive and somatic symptomology might be a promising strategy to reduce children’s physical health issues.

## Limitations

There are some limitations in the current study that must be taken into account when interpreting the results. First, only those mothers of the birth cohort of mother–child dyads have been included for which complete data sets were available and who completed an online survey regarding their own depressive symptomology and their children’s physical health deficits right at the beginning of the pandemic. Their readiness to take part in the survey might reflect a selection bias to talk about these problems openly; and the short time limit for response further restricts the variety of the sample. Additionally, comparison data about the pre-pandemic situation have also been collected retrospectively, whereas the data collection about the actual situation in the pandemic situation was instantly and thus very present and salient. Thus, memory effects could have biased the collected data. Furthermore, the answers to the survey might have been influenced by social desirability. Thus, future studies should be conducted which confirm these results and further elaborate the proposed model using larger and more representative samples.

Second, the sample is untypical in its composition regarding education, socioeconomic status and partnership situation: the majority of the examined mothers (with and without a history of CM) live in a partnership, are highly educated and reported no decreasing income during the pandemic. These factors might be protective against detrimental consequences of CM. Indeed, Meng et al. (2018) [60] have shown a positive relation between higher education and socioeconomic status with psychological well-being and life satisfaction. Likewise, Ford [61] observed more severe trauma-related symptoms for women with a history of victimization if they had a lower educational level. Thus, posttraumatic resilience may be enhanced by education [61]. Also, the experience of positive and successful relationships in adulthood, as it is prevailing in the sample, might buffer a history of adverse experiences [62]. Also, living in a partnership might have attenuated the lack of social support experienced by single parents during the pandemic.

Third, empirical studies have consistently evidenced a link between maternal mental health issues and adverse outcomes for their children. Still, in recent literature the influence not only of maternal, but also paternal mental health problems on children’s emotional, behavioral and adjustment difficulties has been emphasized [63–66]. Thus, for a comprehensive model of interaction also a possible paternal or second caregiver’s psychopathology should be carefully investigated in future studies.

Lastly, the variables collected were not recorded prior to the onset of the pandemic as part of the longitudinal project. Therefore, no comparison of the values to before the pandemic is possible and a bias of the results can therefore not be ruled out. For example, since the beginning of the pandemic studies have already found more than a threefold increase in depressive symptoms [67]. It is possible that the results would not be found at all in a non-pandemic context. Therefore, the present results are not generalizable and are only specifically applicable to the context of a pandemic with severe restrictions on social life.

Future studies should focus on the effects of a pandemic such as SARS-CoV-2 on family systems. Fegert et al. [2] have already shown that disadvantaged and marginalized children and adolescents have been particularly affected by the pandemic. As a result of possible economic recession effects, especially vulnerable families might experience numerous additional stressors, persisting even after the actual pandemic [2]. Still, even by a situation as bad as this pandemic, important lessons can be learned and new innovative ways to support and strengthen young families can be developed and implemented. As the results here show, the attention should focus on a comprehensive and holistic support considering the individual history and background factors of the families. In summary, the findings of this study emphasize the importance of considering a wealth of possibly relevant variables as well as the mechanisms they interact with each other and effect the family system. The current study especially highlighted in detail the associations between CM, maternal mental health as well as somatic symptomology and children’s physical health.

## Conclusion

This study investigated the relationship between CM in mothers and children’s physical health in the extreme situation of a pandemic. In addition, the mediating effects of depressive, somatic, and psychosomatic were examined. The results imply, that maternal experiences of CM can take effect as a risk factor for maternal mental health and children’s physical health in the extreme situation of a pandemic. Maternal CM experiences seem to influence the way how parents deal with stressful situations and increase the risk to suffer depressive and somatic symptoms. The latter impact also their children’s physical health. The results emphasize the significance of a stable and secure family environment for children’s health, especially in times of crisis such as a global pandemic. The mediating effect of the mother’s mental health between CM and the health of the children illustrates a great opportunity of therapy for psychologically stressed parents and for their children, who could also benefit from therapy successes. Therefore, it is important to offer mentally ill parents adapted therapy offers, which they can also perceive and reconcile with their everyday life and their role as parents. Furthermore, services for mentally ill parents should be offered at low thresholds and in everyday situations in order to reduce the hurdle for parents to accept professional help and to reduce the influence of stigmatization and reservations.

## Data Availability

The datasets analyzed as part of the current study are not publicly available due to the current use of the data as part of the ongoing study, but may be requested from the corresponding author upon reasonable request.

## Abbreviations

CM: Childhood maltreatment
CTQ: Childhood Trauma Questionnaire
PHQ-D: Patient Health Questionnaire
